# Disparities in Continuous Glucose Monitoring Among Patients Receiving Care in Federally Qualified Health Centers

**DOI:** 10.1001/jamanetworkopen.2024.45316

**Published:** 2024-11-22

**Authors:** Amisha Wallia, Shivani Agarwal, Andrew L. Owen, Emily L. Lam, Ka’Derricka Davis, Stacy C. Bailey, Sean E. DeLacey, Allison P. Pack, Juan Espinoza, Dana Bright, Alice Eggleston, Eve Walter, Matthew J. O’Brien

**Affiliations:** 1 Division of Endocrinology, Metabolism, and Molecular Medicine, Northwestern University Feinberg School of Medicine, Chicago, Illinois; 2 Chicago Center for Diabetes Translation Research, Chicago, Illinois; 3 Fleischer Institute for Diabetes and Metabolism, Montefiore-Einstein, Bronx, New York; 4 New York-Regional Center for Diabetes Translational Research, Albert Einstein College of Medicine, Bronx; 5 Division of General Internal Medicine, Northwestern Memorial Hospital and Northwestern University Feinberg School of Medicine, Chicago, Illinois; 6 Department of Pediatrics, Northwestern University Feinberg School of Medicine, Chicago, Illinois; 7 Department of Pediatrics, Ann and Robert H. Lurie Children’s Hospital of Chicago, Illinois; 8 AllianceChicago, Illinois; 9 Mount Sinai School of Medicine, New York, New York

## Abstract

**Importance:**

While continuous glucose monitoring (CGM) has been found to improve diabetes care processes and outcomes, adoption remains low.

**Objective:**

To examine the association between CGM prescriptions and individual characteristics among patients with type 1 or 2 diabetes (T1D and T2D, respectively).

**Design, Setting, and Participants:**

Retrospective cross-sectional study using electronic health record data for patients with T1D or T1D from 275 clinic sites nationwide between January 2014 and February 2021. All participating clinics were federally qualified health centers (FQHCs), the largest US system of primary care for vulnerable populations. Data were analyzed from September 2022 to August 2024.

**Main Outcomes and Measures:**

Sociodemographic factors, clinical characteristics, and CGM prescription orders.

**Results:**

A total of 1168 patients with T1D (mean [SD] age, 41.8 [16.0] years; 600 [51.4%] male; 372 [31.9%] Black; 262 [22.4%] Hispanic, and 750 [64.2%] White patients) and 35 216 patients with T2D (mean [SD] age, 58.4 [13.1] years; 19 772 [56.1%] female; 12 030 [34.2%] Black; 12 979 [36.9%] Hispanic, and 20 413 [58.0] White patients) were included. Overall, CGM prescriptions were infrequent (129 [11.0%] for patients with T1D and 362 [1.0%] for those with T2D) but increased throughout the study period. Among patients with T1D, those who reported Hispanic ethnicity (odds ratio [OR], 0.30; 95% CI, 0.16-0.57), Black race (OR, 0.61; 95% CI, 0.38-0.99), or were uninsured (OR, 0.42; 95% CI, 0.23-0.74) had lower multivariable odds of receiving a CGM prescription than White or insured adults, respectively. Similar findings were observed among patients with T2D reporting Hispanic ethnicity (OR, 0.43; 95% CI, 0.32-0.57), Black race (OR, 0.76; 95% CI, 0.59-0.98), or being uninsured (OR, 0.42; 95% CI, 0.31-0.58), relative to their counterparts. Among patients with T2D, hemoglobin A_1c_ values higher than 9.0% (OR, 3.17; 95% CI, 2.37-4.21) and a greater burden of diabetes complications were associated with higher odds of CGM prescription.

**Conclusions and Relevance:**

In this cross-sectional study of electronic health record data, rates of CGM prescription orders were low among FQHC patients with T1D and T2D. Disparities in CGM orders were observed among patients reporting Hispanic ethnicity, Black race, and those who lacked health insurance. Future research is needed to understand the causes of infrequent CGM orders in FQHCs and drivers of observed disparities in this vulnerable patient population.

## Key Points

**Question:**

What is the current state of continuous glucose monitoring (CGM) prescription for type 1 and type 2 diabetes among patients receiving care in federally qualified health centers (FQHCs)?

**Findings:**

In this cross-sectional analysis of electronic health record data from 1168 patients with type 1 diabetes and 35 216 patients with type 2 diabetes receiving primary care in FQHCs, diabetes severity was notable, yet CGM prescription was low. Race, ethnicity, and insurance status were associated with CGM prescription in this vulnerable population.

**Meaning:**

This study suggests low rates of CGM prescription in FQHCs, highlighting a critical need to understand inequities in diabetes technology use in this setting.

## Teaser

This cross-sectional study evaluates the association between continuous glucose monitoring prescriptions and individual characteristics among patients with type 1 and type 2 diabetes receiving care at federally qualified health centers (FQHCs).

## Introduction

Continuous glucose monitoring (CGM) has transformed diabetes care and improved outcomes among patients with type 1 and type 2 diabetes (T1D and T2D).^1^ CGM offers increased precision in diabetes management, improving glycemic control, promoting treatment satisfaction, and enhancing quality of life.^1,2^ There is also evidence that CGM is associated with reduced acute care utilization due to hypo- and hyperglycemic events.^3^ This robust evidence base now informs clinical guidelines recommending CGM use.^4^ Initial studies reported these positive outcomes among patients with T1D, and more recent research demonstrates similar benefits in the larger population of patients with T2D.^3,5^ This prior work also suggests that a greater proportion of patients with T1D have used CGM than their counterparts with T2D.^6,7^

Despite CGM’s potential to improve diabetes care and outcomes, this technology remains underutilized. The uptake of CGM has been greatest in endocrinology clinics, which provide diabetes care for many patients with T1D and more complex cases of T2D.^7,8^ The most recent estimates of CGM use in this setting are as high as 46%.^9,10^ Limited prior research has found lower rates of CGM utilization and wider variation in primary care clinics, where the majority of T2D treatment is provided and the acuity of disease is generally lower.^7,11,12^ Disparities in CGM utilization among racial and ethnic minority groups have been consistently documented across diverse health care settings, highlighting the need to examine health equity implications of this diabetes technology.^9,13,14,15^

Federally qualified health centers (FQHCs) represent the largest national system of primary care clinics for over 30 million medically underserved patients in the US, approximately two-thirds of whom are members of racial or ethnic minority groups with incomes below the federal poverty level.^16^ Almost half of FQHC patients have Medicaid insurance, which constitutes the largest payer in this setting, while approximately one-quarter of patients are uninsured.^17^ While FQHCs represent an important setting to study diabetes health equity, prior research on CGM use in FQHCs has been limited. We sought to describe CGM prescription patterns among patients with T1D and patients with T2D in a large national network of FQHCs, as well as model associations between CGM prescription orders and patient characteristics in this understudied primary care setting.

## Methods

The study protocol was deemed exempt from review and the need for informed consent by the Northwestern University institutional review board because data were deidentified. We conducted a retrospective cross-sectional study of electronic health record (EHR) data between January 2014 and February 2021 from 19 FQHCs comprising 275 clinic sites affiliated with AllianceChicago. This health center–controlled network hosts a centralized EHR infrastructure and a common data warehouse for FQHCs in 21 US states. We included adult patients aged 18 years and older with diabetes, defined with a validated EHR phenotype that included: (1) *International Statistical Classification of Diseases and Related Health Problems, Tenth Revision* diagnostic codes for T1D and T2D; (2) hemoglobin A_1c_ (HbA_1c_; to convert to proportion of total hemoglobin, multiply by 0.01) values 6.5% or higher; random glucose 200 mg/dL or higher (to convert to mmol/L, multiply by 0.0555); and (3) antidiabetic medication prescription.^18^ CGM prescription status was ascertained by the presence of clinicians’ orders for CGM or *Current Procedural Terminology* codes for CGM placement or interpretation. The index date was defined as the first available office visit starting in January 2014, when the first CGM prescription was observed in our data, and ending in February 2021 with the last available data. The reporting of study results adheres to the Strengthening the Reporting of Observational Studies in Epidemiology (STROBE) reporting guideline.^19^

The primary outcome was receipt of the first CGM prescription order or CGM-related procedure code during the study period, which was ascertained using routinely collected EHR data. We analyzed sociodemographic factors and clinical characteristics that were available in the EHR and may be associated with CGM prescription orders. Specifically, these covariates were age, sex, self-reported race and ethnicity (Black, White, and Other [Asian, American Indian or Alaska Native, Native Hawaiian, and Pacific Islander] for race; and Hispanic and non-Hispanic for ethnicity), insurance status at index visit, last available HbA_1c_ value (≤7.0%, 7.1%-8.9%, and ≥9.0%), and the diabetes complication severity index (DCSI) score. The DCSI score and age were analyzed continuously.

### Statistical Analysis

Descriptive statistics were used to examine the sociodemographic and clinical characteristics of patients with T1D and T2D, with stratification by CGM prescription status. We used logistic regression to model the odds of CGM prescription, controlling for all sociodemographic factors and clinical characteristics as potential confounders. The year of the last observed office visit was included in models as a fixed effect for year, accounting for secular trends and any year-specific effects in CGM prescribing during the study period. The odds ratios (ORs) and confidence intervals for all variables in the primary model are shown in eTable 1 in Supplement 1. In a sensitivity analysis, an interaction term in the fully adjusted models for race or ethnicity × HbA_1c_ value was used to explore the potential for racial or ethnic disparities according to participants’ glycemic control. Further sensitivity analyses added adjustment for insulin use, the number of office visits during the study period, and the first observed HbA_1c_ result to the primary model variables (eTable 3 in Supplement 1; descriptive analyses of these additional variables presented in eTable 2 in Supplement 1). eFigures 1 to 3 in Supplement 1 display the rates of CGM prescription orders by race and ethnicity and insurance status. A 2-sided *P* value less than .05 was considered significant for all statistical testing. Analyses were conducted using Stata SE version 18.5 (StataCorp). Data were analyzed from September 2022 to August 2024.

## Results

Among 1168 patients with T1D, the mean (SD) age was 41.9 (16.0) years, 600 (51.4%) were male, 372 (31.9%) were Black, 262 (22%) were Hispanic, and 750 (64.2%) where White (Table 1). Among 35 216 patients with T2D, 19 772 (56.1%) were female, and the mean (SD) age was older (58.4 [13.1] years), with a similar proportion reporting Black race and a higher proportion with Hispanic ethnicity (12 030 [34.2%] Black; 12 979 [36.9%] Hispanic, and 20 413 [58.0%] White patients) (Table 2). Among patients with T1D, 359 (30.7%) were uninsured and 464 (39.8%) had Medicaid insurance, with corresponding proportions in patients with T2D of 14 428 (41.0%) without health insurance and 9602 (27.3%) with Medicaid coverage.

**Table 1.** Characteristics of Patients With Type 1 Diabetes by Continuous Glucose Monitor (CGM) Prescription Status

Characteristic	Patients, No. (%)			*P* value^a^
	Total (N = 1168)	CGM prescription (n = 129)	No CGM prescription (n = 1039)	
Age, mean (SD), y	41.8 (16.0)	38.8 (13.4)	42.2 (16.3)	.02
Race				
Black	372 (31.9)	36 (27.9)	336 (32.3)	.31
White	750 (64.2)	89 (69.0)	661 (63.6)	.23
Other^b^	46 (3.9)	4 (3.1)	42 (4.0)	.60
Ethnicity				
Non-Hispanic	906 (77.6)	116 (89.9)	790 (76.0)	<.001
Hispanic	262 (22.4)	13 (10.1)	249 (24.0)	<.001
Sex				
Male	600 (51.4)	66 (51.2)	534 (51.4)	.96
Female	568 (48.6)	63 (48.8)	505 (48.6)	.96
Insurance status^c^				
Private	256 (21.9)	47 (36.4)	209 (20.1)	<.001
Medicaid	465 (39.8)	52 (40.3)	413 (39.8)	.90
Medicare	88 (7.5)	8 (6.2)	80 (7.7)	.54
Uninsured	359 (30.7)	22 (17.1)	337 (32.4)	<.001
Hemoglobin A_1c_, %^d^				
≤7.0	195 (16.7)	17 (13.2)	178 (17.1)	.51
7.1-8.9	352 (30.1)	39 (30.2)	313 (30.1)	.65
≥9.0	621 (53.2)	73 (56.6)	548 (52.7)	.97
Diabetes Complications Severity Index, mean (SD)	2.2 (4.7)	2.1 (4.3)	2.2 (4.8)	.66

SI Conversion: to convert hemoglobin A_1c_ to proportion of total hemoglobin, multiply by 0.01.

^a^ *P* values are derived from *t* tests of the difference in patient characteristics between those with and without CGM prescriptions during the study period.

^b^ Other racial groups included Asian, American Indian or Alaska Native, Native Hawaiian, and Pacific Islander.

^c^ Insurance status was ascertained at the first visit during the study period.

^d^ Hemoglobin A_1c_ was analyzed as the last available value during the study period.

**Table 2.** Characteristics of Patients With Type 2 Diabetes by Continuous Glucose Monitor (CGM) Prescription Status

Characteristic	Patients, No. (%)			*P* value^a^
	Total (N = 35 216)	CGM prescription (n = 362)	No CGM prescription (n = 34 854)	
Age, mean (SD), y	58.4 (13.1)	57.4 (12.8)	58.4 (13.1)	.15
Race				
Black	12 030 (34.2)	139 (38.4)	11 891 (34.1)	.09
White	20 413 (58.0)	203 (56.1)	20 210 (58.0)	.46
Other^b^	2773 (7.9)	20 (5.5)	2753 (7.9)	.10
Ethnicity				
Non-Hispanic	22 237 (63.1)	285 (78.7)	21 952 (63.0)	<.001
Hispanic	12 979 (36.9)	77 (21.3)	12 902 (37.0)	<.001
Sex				
Male	15 444 (43.9)	166 (45.9)	15 278 (43.8)	.44
Female	19 772 (56.1)	196 (54.1)	19 576 (56.2)	.44
Insurance status^c^				
Private	6347 (18.0)	97 (26.8)	6250 (17.9)	<.001
Medicaid	9602 (27.3)	139 (38.4)	9463 (27.2)	<.001
Medicare	4839 (13.7)	48 (13.3)	4791 (13.8)	.79
Uninsured	14 428 (41.0)	78 (21.6)	14 350 (41.2)	<.001
Hemoglobin A_1c_, %^d^				
≤7.0	13 663 (38.8)	72 (19.9)	13 591 (39.0)	<.001
7.1-8.9	11 402 (32.4)	134 (37.0)	11 268 (32.3)	.08
≥9.0	10 141 (28.8)	156 (43.1)	9995 (28.7)	.001
Diabetes Complications Severity Index, mean (SD)	1.7 (3.6)	2.7 (4.2)	1.7 (3.6)	<.001

SI Conversion: to convert hemoglobin A_1c_ to proportion of total hemoglobin, multiply by 0.01.

^a^ *P* values are derived from *t* tests of the difference in patient characteristics between those with and without CGM prescriptions during the study period.

^b^ Other racial groups included Asian, American Indian or Alaska Native, Native Hawaiian, and Pacific Islander.

^c^ Insurance status was ascertained at the first visit during the study period.

^d^ Hemoglobin A_1c_ was analyzed as the last available value during the study period.

**Figure 1.**
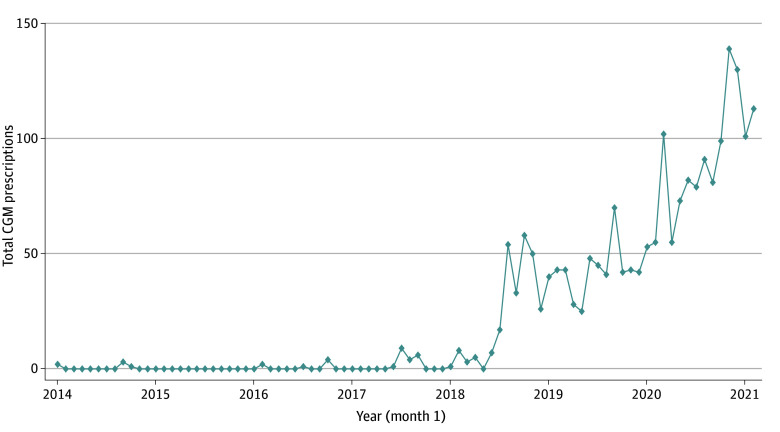
Total Continuous Glucose Monitor (CGM) Prescriptions by Month

The study sample exhibited poor glycemic control, with approximately half of patients with T1D and almost one-third of patients with T2D having HbA_1c_ values above 9.0%. Both patients with T1D and T2D experienced diabetes complications (mean [SD] DSCI scores, 2.2 [4.7] and 1.7 [3.6], respectively). Overall, CGM prescriptions were infrequent (ie, 491 patients [1.3%]), constituting 129 (11.0%) patients with T1D and only 362 (1.0%) patients with T2D. Annual CGM prescriptions increased throughout the study period from only 6 prescriptions in 2014 to 1039 in 2020, and 214 in the first 2 months of 2021. Figure 1 illustrates this trend in CGM prescriptions for the entire cohort, demonstrating very few prescription orders from 2014 to 2018 and then a steady increase beginning in 2019 and continuing through the end of the study period.

**Figure 2.**
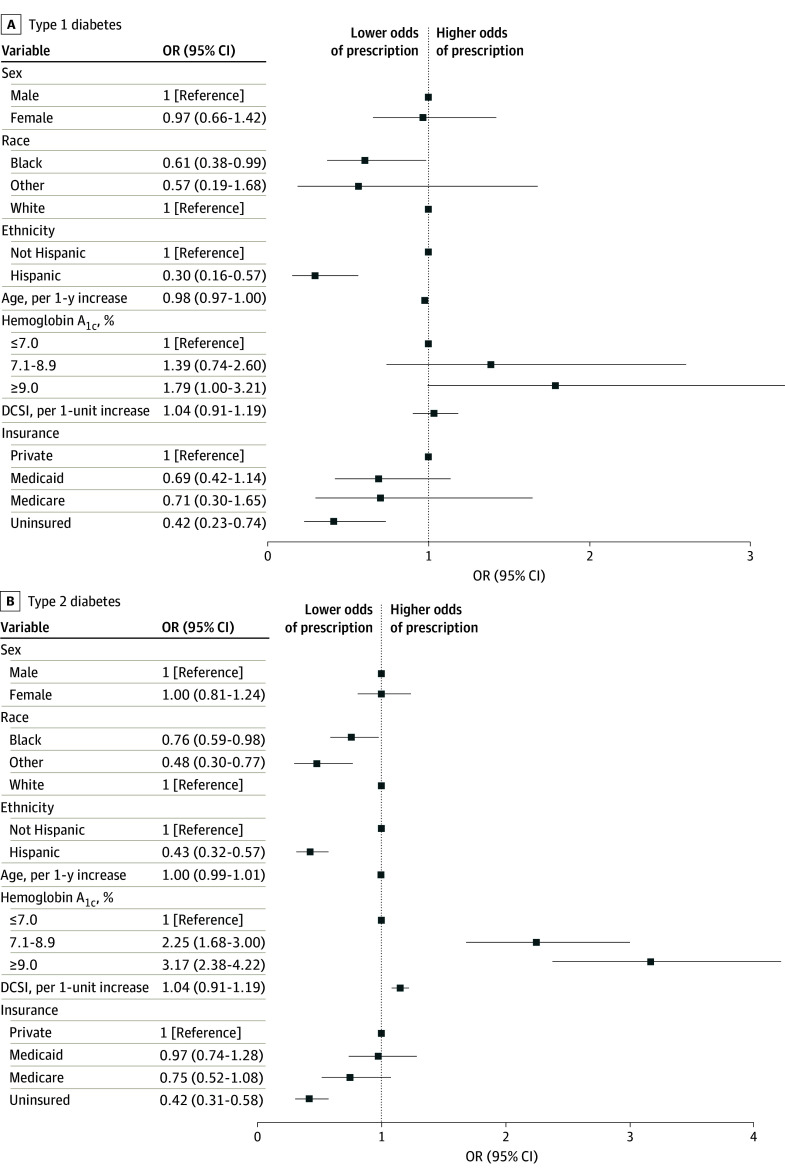
Odds of Continuous Glucose Monitor Prescription Among Patients With Diabetes SI Conversion: to convert hemoglobin A_1c_ to proportion of total hemoglobin, multiply by 0.01. DCSI indicates diabetes complication severity index.

ORs displayed in Figure 2 display the independent associations of selected covariates with CGM prescriptions among patients with T1D and T2D, respectively. ORs and 95% CIs for all covariates included in the primary models are presented in eTable 1 in Supplement 1. Patients with T1D reporting Hispanic ethnicity (OR, 0.30; 95% CI, 0.16-0.57) or Black race (OR, 0.61; 95% CI, 0.38-0.99) exhibited lower odds of CGM prescription than their White counterparts (Figure 2A; eTable 1 in Supplement 1). Similar disparities in CGM prescription were also observed among Hispanic and Black patients with T2D (OR, 0.43; 95% CI, 0.32-0.57 and OR, 0.76; 95% CI, 0.59-0.98, respectively). Lacking health insurance was significantly associated with lower odds of CGM prescription than private insurance coverage among both patients with T1D and T2D (OR, 0.42; 95% CI, 0.23-0.74 and OR, 0.42; 95% CI, 0.31-0.58, respectively). There were no differences in CGM prescription orders by sex in either patients with T1D or T2D.

The level of glycemic control and presence of diabetes complications were significantly associated with CGM prescription orders in patients with T2D. Relative to patients with T2D who had HbA_1c_ values 7.0% or below, the odds of CGM prescription for those with HbA_1c_ of 7.1% to 8.9% were over twice as high and over 3 times as high for those with HbA_1c_ 9.0% or higher. The odds of CGM orders in patients with T2D increased by 15% for every point on the DCSI score. Among patients with T1D, the likelihood of CGM prescription for those with HbA_1c_ values 9.0% or higher compared with those with HbA_1c_ values 7.0% or below did not achieve statistical significance (OR, 1.79; 95% CI, 1.00-3.21). Interaction terms for race and ethnicity × HbA_1c_ values were not statistically significant (data not shown). Sensitivity analyses including insulin prescriptions as a covariate, as well as the number of office visits and the first observed HbA_1c_ value—rather than the last HbA_1c_ value in the primary models—yielded similar findings (eTable 3 in Supplement 1).

## Discussion

This study found low CGM prescription rates among patients receiving diabetes care in FQHCs, the largest national system of safety-net primary care clinics. CGM orders were greater than 10 times more common in the management of T1D than T2D. While infrequent among patients with T2D, CGM orders were significantly associated with poor glycemic control and the presence of diabetes complications. Among both patients with T1D and T2D, we observed significant disparities in CGM prescriptions among those with Black race, Hispanic ethnicity, and no health insurance. It is notable that these disparities exist among patients receiving primary care in FQHCs, which represent the main system of care for historically underserved groups. Our findings highlight the importance of expanding efforts to promote diabetes health equity in this setting.

Disparities in CGM use have also been reported in other health care settings.^20,21^ Most prior research documenting racial and ethnic disparities in CGM use is from academic endocrinology clinics,^9,13,15,22^ with a recent study reporting similar findings in Veterans Affairs (VA) outpatient clinics.^14^ Our study is the first that we know of to demonstrate racial and ethnic CGM disparities in a national network of FQHCs, where there are additional resources available to support the diabetes care of the vulnerable patient populations they serve.^23^ This may explain why the magnitude of disparities we observed among Black patients in FQHCs was smaller than those reported in other settings.^13,15^ The persistence of racial and ethnic disparities in CGM use across diverse health care settings suggests that intractable forces such as limited access to care and medical devices, implicit bias, and medical mistrust may play a role.^24^ Some prior efforts have demonstrated promise in reducing disparities in CGM uptake, including a Colorado Medicaid program that provided full subsidies for CGM devices.^25^ Results from this program suggest that financial barriers remain significantly associated with disparities in CGM use.

CGM uptake has expanded significantly over the last 2 decades, first in T1D and increasingly in T2D. The earliest adoption of CGMs occurred in endocrinology clinics, where less than 7% of antidiabetic medication prescriptions are written.^12^ New clinical trial data supports CGM use in primary care,^1,26^ providing an evidence base to justify the growth of this technology among a much larger diabetic population. Employing CGM effectively across outpatient settings will require overcoming systemic barriers including a lack of time, support, and expertise among clinicians.^27,28^ Recently, the Federal Drug Administration recently approved the first over-the-counter CGM,^29^ signaling a trend that may expand the market significantly. While this may promote increased CGM access, with the potential to reduce disparities in CGM use, new challenges will emerge as patients with diabetes may use these devices with less support from their health care clinicians.

### Strengths and Limitations

Strengths of our analysis include the novelty of studying CGM orders among patients receiving their primary care in FQHCs. This represents an important and understudied setting for exploring diabetes health equity given the high proportion of patients from racial and ethnic minority groups and those who are uninsured or underinsured. The prevalence of uncontrolled diabetes in FQHCs, reported among 35.6% of patients nationwide,^30^ is higher than that observed overall among US adults with diabetes.^31^ This observation also underscores the importance of examining CGM prescriptions in FQHCs, given the potential of this diabetes technology to improve glycemic control among the large and particularly vulnerable patient populations they serve. While some prior studies of CGM use among FQHC patients have focused on samples from a single FQHC or a single state, our data are derived from a national FQHC network spanning 275 clinic sites in 19 US states.^25,32^

Our study also has notable limitations. CGM orders observed in the EHR are not equivalent to long-term use, which cannot be verified with our dataset because it does not include pharmacy claims. FQHCs offer primary care for medically underserved patients, which does not include subspecialty care by endocrinologists, who are more likely to prescribe CGM.^8,33^ The SARS-CoV-2 pandemic occurred during the latter part of our study period, when telemedicine visits became common.^34^ The study data did not include information about telehealth visits, when CGM prescriptions may have been less likely than in-person visits. However, we observed a steady increase in CGM prescriptions from 2018 to the end of the study period in 2021, and our models included a fixed effect for year that adjusted for changes in care delivery during the SARS-CoV-2 pandemic. While we adjusted for the number of office visits, we could not determine whether they were primarily associated with diabetes management. In addition, we could not adjust for state-specific trends in CGM prescribing that may have impacted our findings. Since the end of the study period in February 2021, insurance coverage for CGM has expanded, out-of-pocket costs for CGM have decreased, and primary care clinicians have become increasingly familiar with this diabetes technology. Investigation of more recent FQHC data is needed to confirm whether low use of CGMs and the CGM-related disparities reported in this study persist.

## Conclusions

In this cross-sectional study, we highlighted a significant gap in implementing CGM clinical practice guidelines in FQHCs.^4^ We observed infrequent use of CGMs in FQHCs relative to prior data from other ambulatory settings. In addition, we reported persistent disparities in CGM prescriptions among racial and ethnic minorities, as well as those who were uninsured. Future research is needed to understand barriers to CGM use among FQHC clinicians and their patients. Prior research suggests that eliminating financial barriers significantly increases CGM uptake, while reducing racial and ethnic disparities.^25,32^ Changes in Medicaid policy have the greatest potential to improve CGM use in FQHCs, as it is the primary payer in this setting.^17^ However, additional coverage strategies and alternate mechanisms for accessing CGM will be needed given the large proportion of uninsured patients who receive primary care in FQHCs.

## Notes

Supplement 1. **eTable 1. **Multivariable Association of Patient Characteristics and Continuous Glucose Monitor Prescription (Primary Model) **eTable 2.** Descriptive Statistics of Additional Variables in Supplementary Logistic Regression Analysis **eTable 3. **Multivariable Association of Patient Characteristics and Continuous Glucose Monitor Prescription with Additional Variables **eFigure 1. **CGM Prescriptions by Month and Ethnicity **eFigure 2. **CGM Prescriptions by Month and Insurance Status **eFigure 3. **CGM Prescriptions by Month and Race

Supplement 2.
Data Sharing Statement
