# Supplementary material for: Disparities in Continuous Glucose Monitoring Among Patients Receiving Care in Federally Qualified Health Centers
Source: JAMA Netw Open. 2024 Nov 22;7(11):e2445316. doi: 10.1001/jamanetworkopen.2024.45316 (PMC11584923; doi:10.1001/jamanetworkopen.2024.45316)
Supplement: Supplement 1. — eTable 1. Multivariable Association of Patient Characteristics and Continuous Glucose Monitor Prescription (Primary Model) eTable 2. Descriptive Statistics of Additional Variables in Supplementary Logistic Regression Analysis eTable 3. Multivariable Association of Patient Characteristics and Continuous Glucose Monitor Prescription with Additional Variables eFigure 1. CGM Prescriptions by Month and Ethnicity eFigure 2. CGM Prescriptions by Month and Insurance Status eFigure 3. CGM Prescriptions by Month and Race [file jamanetwopen-e2445316-s001.pdf]

Supplemental Online Content

Wallia A, Agarwal S, Owen AL, et al. Disparities in continuous glucose monitoring among patients in federally qualified health centers. *JAMA Netw Open*. 2024;7(11):e2445316. doi:10.1001/jamanetworkopen.2024.45316

eTable 1. Multivariable Association of Patient Characteristics and Continuous Glucose Monitor Prescription (Primary Model)

eTable 2. Descriptive Statistics of Additional Variables in Supplementary Logistic Regression Analysis

eTable 3. Multivariable Association of Patient Characteristics and Continuous Glucose Monitor Prescription with Additional Variables

eFigure 1. CGM Prescriptions by Month and Ethnicity

eFigure 2. CGM Prescriptions by Month and Insurance Status

eFigure 3. CGM Prescriptions by Month and Race

This supplemental material has been provided by the authors to give readers additional information about their work.

© 2024 Wallia A et al. *JAMA Network Open*.

eTable 1. Multivariable Association of Patient Characteristics and Continuous Glucose Monitor Prescription (Primary Model)

Variable	Patients with Type 1 Diabetes (n=1,168)	Patients with Type 2 Diabetes (n=35,216)
	OR (95% CI)	OR (95% CI)
Sex		
Male	REF	REF
Female	0.97 (0.66 – 1.42)	1.00 (0.81 – 1.24)
Race		
White	REF	REF
Black	0.61 (0.38 – 0.99)	0.76 (0.59 – 0.98)
Other	0.57 (0.19 – 1.68)	0.48 (0.30 – 0.77)
Ethnicity		
Non-Hispanic	REF	REF
Hispanic	0.30 (0.16 – 0.57)	0.43 (0.32 – 0.57)
Age, years (continuous)	0.98 (0.97 – 1.00)	1.00 (0.99 – 1.01)
A1c, %		
≤ 7.0	REF	REF
7.1 – 8.9	1.39 (0.74 – 2.60)	2.25 (1.68 – 3.00)
≥ 9.0	1.79 (1.00 – 3.21)	3.17 (2.38 – 4.22)
Diabetes Complications Severity Index (continuous)	1.04 (0.91 – 1.19)	1.15 (1.08 – 1.22)
Insurance		
Private	REF	REF
Medicaid	0.69 (0.42 – 1.14)	0.97 (0.74 – 1.28)
Medicare	0.71 (0.30 – 1.65)	0.75 (0.52 – 1.08)
Uninsured	0.42 (0.23 – 0.74)	0.42 (0.31 – 0.58)
Year of Last Visit		
2015	(empty)	REF
2016	REF	0.85 (0.05 – 13.61)
2017	(empty)	1.50 (0.14 – 16.57)
2018	1.20 (0.11 – 13.58)	3.24 (0.38 – 27.80)
2019	3.04 (0.37 – 24.96)	16.44 (2.24 – 120.57)
2020	9.93 (1.34 – 73.42)	26.81 (3.74 – 191.99)
2021	16.22 (2.19 – 120.09)	41.63 (5.82 – 297.61)

OR = Odds Ratio

© 2024 Wallia A et al. *JAMA Network Open*.

eTable 2. Descriptive Statistics of Additional Variables in Supplementary Logistic Regression Analysis

Variable	Patients with Type 1 Diabetes (n=1,168)	Patients with Type 2 Diabetes (n=35,216)
	n (%)	n (%)
First Observed A1c Value in Study Period, %		
≤7.0	167 (14.3)	14,190 (40.3)
7.1 – 8.9	342 (29.3)	9,568 (27.2)
≥ 9.0	659 (56.4)	11,458 (32.5)
Number of Office Visits during Study Period		
1-4	278 (24.0)	6,919 (19.7)
5-8	264 (23.0)	7,014 (19.9)
9-14	247 (21.2)	7,198 (20.4)
15-24	209 (17.9)	7,382 (21.0)
25+	170 (14.6)	6,703 (19.0)
Insulin Prescription during Study Period		
Yes	1,092 (93.5)	11,672 (33.1)
No	76 (6.5)	23,544 (66.9)

© 2024 Wallia A et al. *JAMA Network Open*.

eTable 3. Multivariable Association of Patient Characteristics and Continuous Glucose Monitor Prescription with Additional Variables

Variable	Patients with Type 1 Diabetes (n=1,168)	Patients with Type 2 Diabetes (n=35,216)
	OR (95% CI)	OR (95% CI)
Sex		
Male	REF	REF
Female	1.01 (0.68 – 1.49)	0.90 (0.73 – 1.12)
Race		
White	REF	REF
Black	0.63 (0.39 – 1.03)	0.75 (0.58 – 0.97)
Other	0.55 (0.19 – 1.65)	0.55 (0.34 – 0.88)
Ethnicity		
Non-Hispanic	REF	REF
Hispanic	0.31 (0.16 – 0.58)	0.46 (0.34 – 0.62)
Age, years (continuous)	0.98 (0.96 – 0.99)	1.00 (0.99 – 1.01)
First A1c value, %		
≤ 7.0	REF	REF
7.1 – 8.9	1.50 (0.78 – 2.89)	1.35 (1.01 – 1.79)
≥ 9.0	1.46 (0.80 – 2.66)	1.28 (0.97 – 1.69)
Diabetes Complications Severity Index (continuous)	1.02 (0.89 – 1.17)	1.03 (0.97 – 1.10)
Insulin Prescription	9.00 (1.20 – 67.79)	4.76 (3.68 – 6.15)
Insurance		
Private	REF	REF
Medicaid	0.73 (0.44-1.20)	0.84 (0.64 – 1.12)
Medicare	0.72 (0.30 – 1.69)	0.68 (0.47 – 0.98)
Uninsured	0.44 (0.24 – 0.78)	0.42 (0.31 – 0.58)
Year of Last Visit		
2015	(empty)	REF
2016	REF	0.81 (0.05 – 12.90)
2017	(empty)	1.39 (0.13 – 15.36)
2018	1.06 (0.09 – 12.06)	2.94 (0.34 – 25.26)
2019	2.62 (0.32 – 21.67)	13.82 (1.88 – 101.72)
2020	7.41 (0.99 – 55.38)	21.82 (3.03 – 157.03)
2021	11.80 (1.57 – 88.82)	31.24 (4.34 – 224.87)
Number of Visits		
1-4 Visits	REF	REF
5-8 Visits	1.34 (0.67 – 2.70)	1.24 (0.79 – 1.95)
9-14 Visits	1.65 (0.84 – 3.25)	1.19 (0.77 – 1.85)
15-24 Visits	2.41 (1.22 – 4.73)	1.34 (0.88 – 2.04)
25+	1.47 (0.69 – 3.14)	1.87 (1.25 – 2.81)

* Additional variables are number of visits, first A1c value, and insulin prescription

© 2024 Wallia A et al. *JAMA Network Open*.

eFigure 1. CGM Prescriptions by Month and Ethnicity

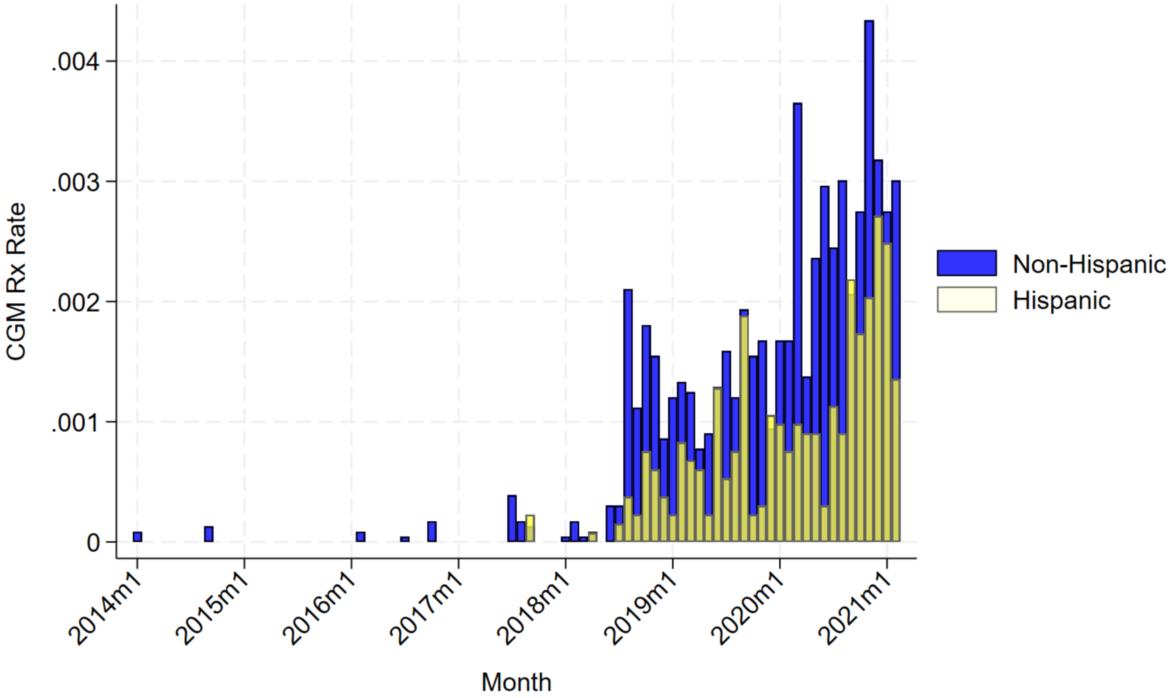

© 2024 Wallia A et al. *JAMA Network Open*.

eFigure 2. CGM Prescriptions by Month and Insurance Status

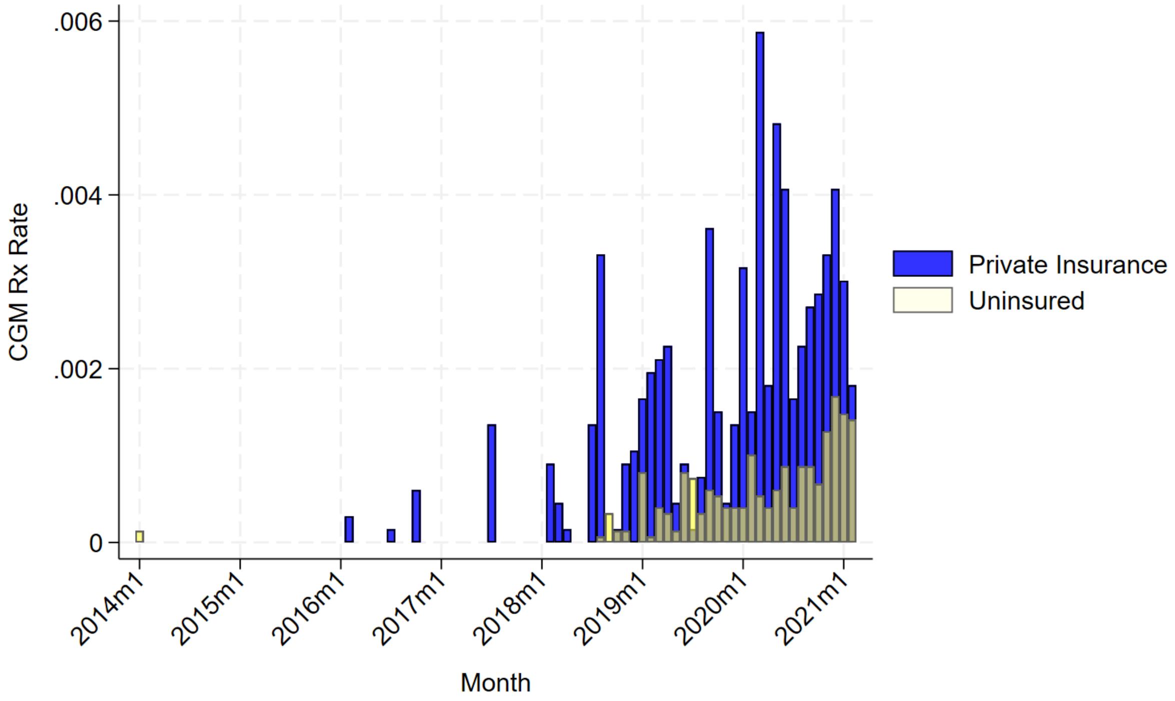

© 2024 Wallia A et al. *JAMA Network Open*.

eFigure 3. CGM Prescriptions by Month and Race

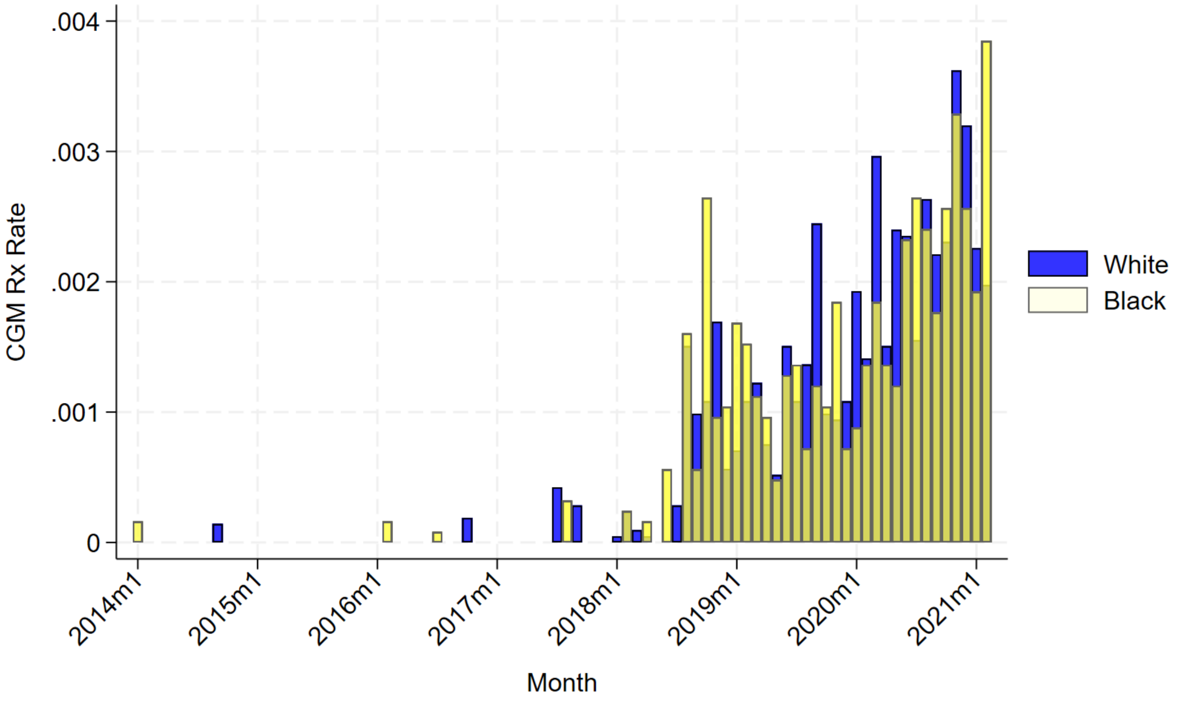

© 2024 Wallia A et al. *JAMA Network Open*.
