# Supplementary material for: Disparities in Continuous Glucose Monitoring Among Patients Receiving Care in Federally Qualified Health Centers
Source: JAMA Netw Open. 2024 Nov 22;7(11):e2445316. doi: 10.1001/jamanetworkopen.2024.45316 (PMC11584923; doi:10.1001/jamanetworkopen.2024.45316)
Supplement: Supplement 2. — Data Sharing Statement [file jamanetwopen-e2445316-s002.pdf]

Data Sharing Statement

Wallia. Disparities in Continuous Glucose Monitoring Among Patients in Federally Qualified Health Centers. *JAMA Netw Open*. Published November 15, 2024.
doi:10.1001/jamanetworkopen.2024.45316

Data
Data available: Yes
Data types: Other (please specify)
Additional Information: Per institutional policy
How to access data: upon request from a-wallia@northwestern.edu, per institutional policy
When available: With publication

Supporting Documents
Document types: None

Additional Information
Who can access the data: upon request, per institutional policy
Types of analyses: per institutional policy
Mechanisms of data availability: per institutional policy
